# *GST*-*omega* genes interact with environmental tobacco smoke on adult level of lung function

**DOI:** 10.1186/1465-9921-14-83

**Published:** 2013-08-09

**Authors:** Kim de Jong, H Marike Boezen, Nick HT ten Hacken, Dirkje S Postma, Judith M Vonk

**Affiliations:** 1 University of Groningen, University Medical Center Groningen (UMCG), Department of Epidemiology, Groningen, the Netherlands; 2 University of Groningen, University Medical Center Groningen (UMCG), Department of Pulmonology, Groningen, the Netherlands; 3 University Medical Center Groningen, Groningen Research Institute for Asthma and COPD (GRIAC), Groningen, the Netherlands

**Keywords:** Genes, Environmental tobacco smoke, Lung function

## Abstract

**Background:**

Lung growth *in utero* and lung function loss during adulthood can be affected by exposure to environmental tobacco smoke (ETS). The underlying mechanisms have not been fully elucidated. Both ETS exposure and single nucleotide polymorphisms (SNPs) in *Glutathione S*-*Transferase* (*GST*) *Omega* genes have been associated with the level of lung function. This study aimed to assess if *GSTO* SNPs interact with ETS exposure *in utero* and during adulthood on the level of lung function during adulthood.

**Methods:**

We used cross-sectional data of 8,128 genotyped participants from the LifeLines cohort study. Linear regression models (adjusted for age, sex, height, weight, current smoking, ex-smoking and packyears smoked) were used to analyze the associations between *in utero*, daily and workplace ETS exposure, *GSTO* SNPs, the interaction between ETS and *GSTO*s, and level of lung function (FEV_1_, FEV_1_/FVC). Since the interactions between ETS and *GSTO*s may be modified by active tobacco smoking we additionally assessed associations in never and ever smokers separately. A second sample of 5,308 genotyped LifeLines participants was used to verify our initial findings.

**Results:**

Daily and workplace ETS exposure was associated with significantly lower FEV_1_ levels. *GSTO* SNPs (recessive model) interacted with *in utero* ETS and were associated with higher levels of FEV_1_, whereas the interactions with daily and workplace ETS exposure were associated with lower levels of FEV_1_, effects being more pronounced in never smokers. The interaction of *GSTO2* SNP rs156697 with *in utero* ETS associated with a higher level of FEV_1_ was significantly replicated in the second sample. Overall, the directions of the interactions of *in utero* and workplace ETS exposure with the SNPs found in the second (verification) sample were in line with the first sample.

**Conclusions:**

*GSTO* genotypes interact with *in utero* and adulthood ETS exposure on adult lung function level, but in opposite directions.

## Background

Lung function loss is common in chronic respiratory diseases like chronic obstructive pulmonary disease (COPD), cystic fibrosis (CF) and interstitial lung fibrosis, and associates with all-cause and other specific mortality [1,2]. Both environmental and genetic factors contribute to lung function loss. Active cigarette smoking is regarded as the most important environmental risk factor, yet other factors exist. Like active smoking, passive cigarette smoking or environmental tobacco smoke (ETS) exposure induces inflammation and oxidative stress in the lungs [3]. ETS exposure has been associated with reduced level of lung function at birth [4,5] and in adulthood [6,7], as well as with respiratory symptoms [8,9] and increased COPD risk [10,11]. In other words, ETS exposure can affect *in utero* lung development, lung growth during childhood and lung function loss during adulthood. However, the underlying mechanisms have not been elucidated. Furthermore, these underlying mechanism are not necessarily similar for ETS exposure *in utero* and during adulthood given the fact that both the mode of exposure and the period of exposure within the life-span are different.

Although it has been very well established that genetic factors contribute to lung function level [12], less is known about how genetic factors modify effects of ETS exposure on the level of lung function during the life-span. Glutathione S-Transferases (GSTs) are a family of enzymes involved in the detoxification of xenobiotic substances such as tobacco smoke, and play an essential role in oxidative stress reactions [13,14]. Polymorphisms in the *GST*-*mu*, -*pi*, and -*theta* genes have been described to interact with tobacco smoke exposure with respect to asthma development and atopy in asthmatic children [15,16] and lower childhood level of lung function [17]. The *GST*-*omega* (*GSTO*) class has been less well studied. Of interest, GSTO enzymes have thioltransferase activity and can catalyze specific reduction reactions with compounds that are not substrates for other GSTs, suggesting an important role for GSTO in oxidative stress reactions [18,19] and in biotransformation of inorganic arsenic [20], a component present in tobacco smoke. GSTO1 has also been reported to activate IL-1β [21], a cytokine that is important for tobacco smoke induced inflammation and fibrosis [22,23]. Harju et al. showed that GSTO1 is abundantly expressed in alveolar macrophages [24]. Furthermore, a genome wide association analysis in the Framingham Heart Study found a *GSTO2* SNP (rs156697) to be associated with both lower level of FEV_1_ and FVC [12]. Another study could not replicate this association between rs156697 and FEV_1_, but found an association with COPD, defined by lower lung function [25]. It is unknown whether *GSTO1* and *GSTO*2 SNPs modify effects of ETS exposure on the level of lung function.

This study aimed to assess if *GSTO* SNPs interact with *in utero* and/or adulthood ETS exposure on lung function level in a general population.

## Methods

### Study sample and measurements

We included 8,128 genetically unrelated individuals from the LifeLines cohort study. The LifeLines cohort is designed to investigate universal risk factors and their modifiers for multifactorial chronic diseases and comorbidities [26]. All subjects received a questionnaire and underwent a medical examination including collection of a blood sample for DNA extraction. The questionnaire included questions regarding personal characteristics, smoking habits and ETS exposure. We used self-reported *in utero* ETS exposure (coded as: no/yes/do not know), daily ETS exposure based on self-reported hours of exposure to other person’s tobacco smoke per day (coded as: <1/≥1 hour per day), and ETS exposure at work (answer categories: no/yes/not applicable). The medical examination included spirometry (FEV_1_ and FEV_1_/FVC) performed in a standardized setting following ATS guidelines using a Welch Allyn Version 1.6.0.489, PC-based SpiroPerfect with CardioPerfect Workstation software. A second sample, including 5,308 individuals from the LifeLines cohort study genotyped at a later stage, was used to verify our initial findings. Questionnaires, medical examinations and genotyping at baseline were performed according to the same standardized protocol in sample 1 and sample 2.

### Genotyping

Genotyping was performed using IlluminaCytoSNP-12 arrays. Beagle (version 3.3) and the HapMap3-database were used to impute additional SNPs. Three Haplotype-tagging SNPs in the *GSTO1*-*2* cluster with minor allele frequency (MAF) > 0.1, HW-equilibrium p-value > 0.05, and R^2^ < 0.8 were selected with Haploview (version 4.2). We additionally included SNP rs156697 that was associated with lower FEV_1_ and FVC in the Framingham Heart Study [12]. The four selected SNPs were rs4925, rs1147611, rs156697 and rs156699. LD-plot (Additional file 1: Figure S1) and genotype frequencies (Additional file 1: Table S1) are presented in the online supplement.

### Statistical analysis

Linear regression models adjusted for age, sex, height, weight, current smoking, ex-smoking, and packyears smoked, were used to analyze the associations between ETS exposure, *GSTO* SNPs, the interaction between ETS and *GSTO*s and level of lung function (FEV_1_, FEV_1_/FVC). Since the interactions between ETS and *GSTO*s may be modified by active tobacco smoking we additionally assessed associations in never and ever smokers separately. All analyses were performed using SPSS version 20.0 (IBM Corporation, USA). P-values < 0.05 (tested 2-sided) were considered statistically significant. To examine the robustness of our findings we used False Discovery Rate (FDR) correction for multiple testing [27], taking into account the number of tests performed for each of the exposures (4 SNPs * 3 separate analyses (all/never/ever) * 2 outcomes (FEV_1_, FEV_1_/FVC)).

### Ethical approval

The study was approved by the Medical Ethics Committee of the University Medical Center Groningen, Groningen, The Netherlands (ref. METc 2007/152).

## Results

### Population characteristics

Characteristics of both samples 1 and 2 are shown in Table 1. Briefly, both samples included more females than males and more ever than never smokers. 15% of the participants did not know whether their mother smoked during pregnancy, and this group was excluded in the analyses on the effect of *in utero* exposure on the level of lung function. Of the remaining participants, 13% reported *in utero* ETS exposure. Almost 25% of the participants reported daily ETS exposure (≥1 hours), and 7% reported ETS exposure at the workplace (in 12% of the participants this was not applicable because of unemployment; this group was coded as a separate category in the analyses). The median level of self-reported exposure was 2 hours per day within the group with daily ETS exposure (25th percentile = 1 hour, 75th percentile = 4 hours).

**Table 1:** **Characteristics participants included in sample 1 and sample** 2

	**Sample 1**	**Sample 2 (verification)**
**n**	8128	5308
**Males**, n (%)	3483 (43)	2133 (40)
**Age**, median (min-max)	47 (18–89)	48 (21–90)
**Smoking status**, n (%)		
Never, n (%)	3277 (40)	2154 (41)
Ex, n (%) [median (min-max)]	2882 (36) [8 (0–86)]	2014 (39) [7 (0–100)]
Current, n (%) [median (min-max)]	1936 (24) [15 (0–100)]	1065 (20) [16 (0–81)]
**ETS exposure**, n (%)		
*In utero*	867 (13)	559 (13)
≥ 1 hour/day	1788 (24)	1029 (21)
At the workplace	565 (7)	303 (6)
**Lung function**, mean (sd)		
FEV_1_ (ml)	3412 (831)	3331 (840)
FEV_1_pp (%)^1^	102 (14)	102 (14)
FEV_1_/FVC (%)	77 (7)	76 (7)
**Spirometry available**, n	7635	5070

The second sample (sample 2) was used to verify the initial findings from sample 1, both samples are part of the LifeLines population-based cohort study.

^1^ FEV_1_pp = FEV_1_ as percentage predicted based on reference equations constructed by Quanjer et al. [28].

### ETS exposure and level of lung function

**Figure 1:**
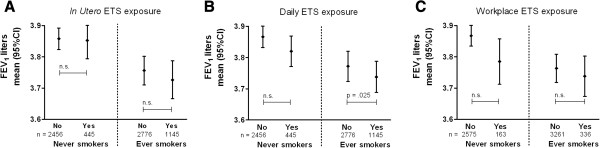
**Mean FEV**_**1 **_**(liters) for non**-**exposed and exposed subjects stratified by smoking status (never/ever smoker).** The analysis was adjusted for sex, current smoking, packyears smoked, and centered for group specific (never/ever smokers) means for age, height and weight. **A**: *In utero* ETS (no/yes). **B**: daily ETS exposure (</≥1 hr). **C**: ETS exposure at the workplace (no/yes).

Complete data on all covariates was available for n = 6003, 6822 and 7149 subjects for *in utero* ETS exposure (excluding ‘do not know’), daily and workplace ETS exposure respectively. *In utero* ETS exposure was not associated with FEV_1_ and was negatively associated with FEV_1_/FVC [b = −0.6% (95% CI = −1.1 ; -0.1)]. The association with FEV_1_/FVC was similar for never and ever smokers (Additional file 1: Table S2). Daily ETS exposure (≥1 hour) was significantly associated with lower FEV_1_ [−37 ml (−65; -8)], and not with FEV_1_/FVC. Workplace ETS exposure was significantly associated with lower FEV_1_ [−43 ml (−86; 0)], and FEV_1_/FVC [−0.6% (−1.2 ; 0)]. Stratification by smoking status resulted in significant associations of ETS exposure with FEV_1_ in never smokers only, effect estimates being −45 ml (−91 ; 0) and −82 ml (−153 ; -11) for daily and workplace ETS respectively (Figure 1). Daily and workplace ETS exposure were not significantly associated with FEV_1_/FVC in never or ever smokers (for all effect estimates see Additional file 1: Table S2).

### SNPs and level of lung function

Subjects heterozygous for SNP rs4925 had a significantly higher FEV_1_ [23 ml (0 ; 45)] and subjects heterozygous for rs156699 a significantly higher FEV_1_/FVC [0.3% (0 ; 0.7)] than wild types. There were no other significant associations between genotype and lung function (Additional file 1: Table S3).

### Effect of interaction between *GSTO* SNPs and *in utero* ETS exposure on level of lung function

**Figure 2:**
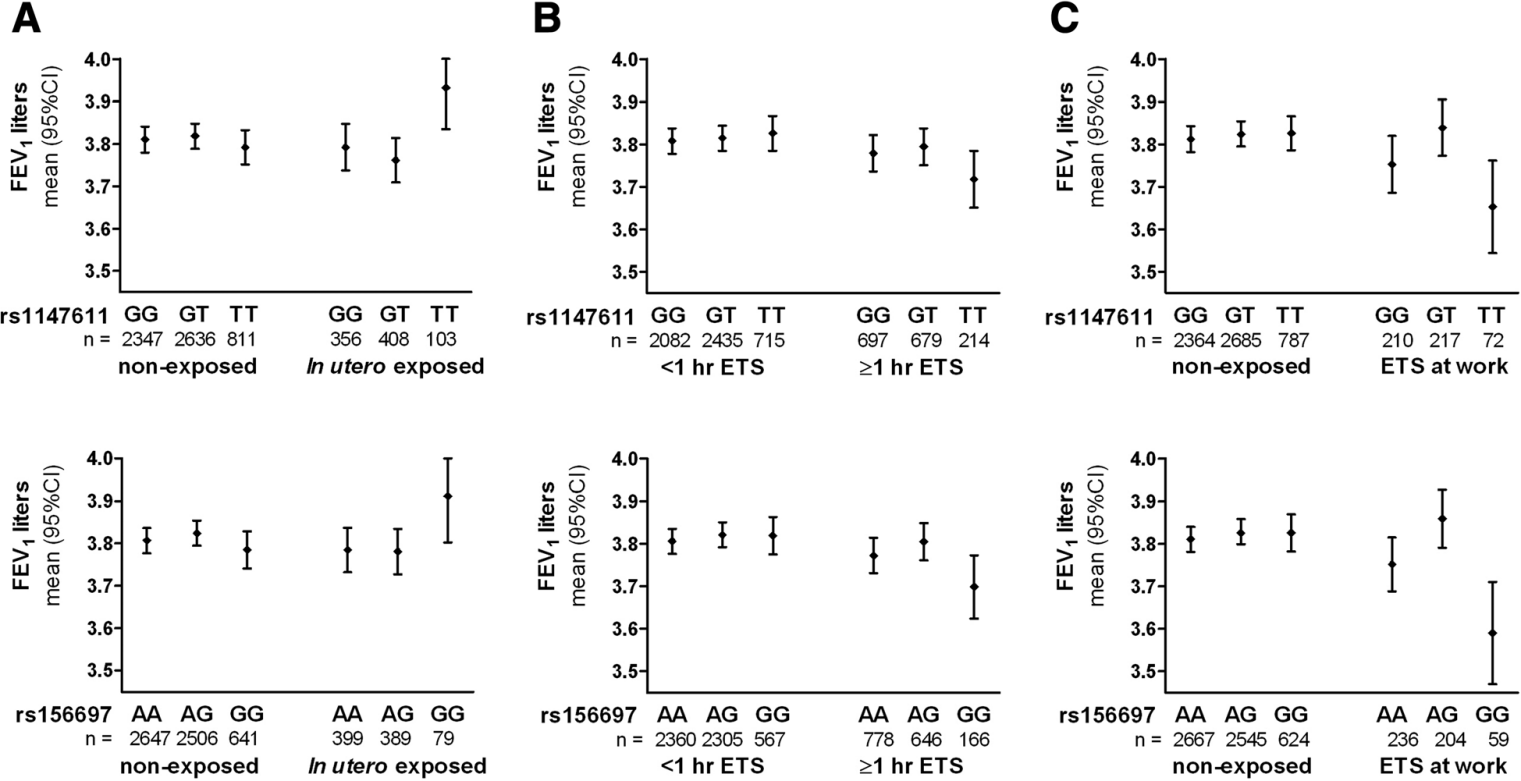
**Mean FEV**_**1 **_**(liters) for non**-**exposed and ETS exposed subjects stratified by genotype for the two genotyped SNPs rs1147611 and rs156699.***GSTO1* SNP rs1147611 (upper row) and *GSTO2* SNP rs156699 (lower row). The analysis was adjusted for sex, current, ex-smoking, packyears smoked, and centered for mean age, height and weight. **A**: *In utero* ETS (no/yes). **B**: daily ETS exposure (</≥1 hr). **C**: ETS exposure at the workplace (no/yes).

Mean FEV_1_ levels were significantly different (i.e. higher with *in utero* ETS, and lower with daily and workplace ETS exposure) in subjects carrying both minor alleles for all four *GSTO* SNPs compared to wild type and heterozygote genotypes (Figure 2). Therefore we used a recessive genetic model in subsequent analyses. *In utero* ETS exposure interacted with all four *GSTO* SNPs and these interactions were associated with higher level of FEV_1_ level (Table 2), i.e. being homozygote for the minor alleles was associated with a higher FEV_1_ only in subjects that were exposed to ETS *in utero*. There was no association with FEV_1_/FVC (Additional file 1: Table S4). Associations were more pronounced in never smokers, except for SNP rs156697 (Table 2). Most of these interactions remained significant after FDR correction for multiple testing.

**Table 2:** **Effects for *in utero *ETS exposure** (**no**/**yes**), **the SNPs**, **and the interaction of *GSTO *SNPs** (**recessive model**) **with *in utero *ETS exposure on FEV**_**1**_

			**FEV1 (ml) b (95% CI)**	
**Gene**	**Variable**	**All**	**Never smokers**	**Ever smokers**
	N, in analysis	6003	2576	3427
*GSTO1*	*In utero* ETS	−33 (−69 ; 4)	−25 (−81 ; 32)	−43 (91 ; 5)
	rs4925	−33 (−77 ; 10)	−1 (65 ; 63)	−58 (−117 ; 1)
	ETS*rs4925	^$^**177** (**50** ; **305**)**	**196** (**15** ; **376**)*	161 (−18 ; 340)
*GSTO1*	*In utero* ETS	−**40** (−**77** ; -**2**)*	−32 (−89 ; 26)	−**51** (−**100** ; -**2**)*
	rs1147611	−22 (−58 ; 14)	6 (−46 ; 58)	−46 (−96 ; 3)
	ETS*rs1147611	^$^**177** (**72** ; **283**)***	^$^**206** (**47** ; **365**)*	**166** (**25** ; **307**)*
*GSTO2*	*In utero* ETS	−**41** (−**78** ; -**3**)*	−28 (−85 ; 30)	−**55** (−**104** ; -**6**)*
	rs156697	−28 (−64 ; 9)	9 (−44 ; 62)	−**59** (−**110** ; -**8**)*
	ETS*rs156697	^$^**198** (**90** ; **307**)***	**181** (**20** ; **342**)*	^$^**219** (**72** ; **366**)**
*GSTO2*	*In utero* ETS	−33 (−70 ; 3)	−24 (−81 ; 32)	−45 (−93 ; 4)
	rs156699	−30 (−70 ; 10)	−9 (−66 ; 49)	−48 (−104 ; 7)
	ETS*rs156699	^$^**160** (**41** ; **278**)**	**193** (**12** ; **374**)*	143 (−15 ; 300)

The linear regression model for the whole group was adjusted for sex, age, height, weight, current, ex-smoking and packyears smoked. Consequently we stratified by smoking status (never/ever) and adjusted for the other possible confounders.

*p-value<0.05 **p-value<0.01 *** p-value<0.001.

^$^significant after FDR correction for multiple testing.

### Effect of interaction between *GSTO* SNPs and adulthood ETS exposure on level of lung function

Daily ETS exposure (≥1 hour) interacted significantly with SNPs rs4925, rs1147611 and rs156699 and these interactions were associated with lower FEV_1_ level (Table 3). Workplace ETS interacted significantly with all four SNPs and these interactions were associated with lower FEV_1_ (Table 4). In other words, being homozygote for the minor alleles of the *GSTO* SNPs was associated with lower level of lung function only in subjects that were exposed to daily and workplace ETS exposure. Stratification by smoking status showed that the negative interaction effects between ETS exposure and the SNPs on FEV_1_ level were consistently more pronounced in never smokers (Tables 3 and 4). No significant interactions were found between daily and workplace ETS exposure and the *GSTO* SNPs on FEV_1_/FVC in the whole group, or when stratified by smoking status (never/ever) (Additional file 1: Tables S5 and S6). All significant interactions of the *GSTO* SNPs with workplace ETS on level of FEV_1_ remained significant after FDR correction for multiple testing, the interactions with daily ETS exposure did not remain significant.

**Table 3:** **Effects for daily ETS exposure** (</≥**1 hr**), **the SNPs**, **and the interaction of *GSTO *SNPs** (**recessive model**) **with daily ETS exposure on FEV**_**1**_

			**FEV**_**1 **_**(ml) b (95% CI)**	
**Gene**	**Variable**	**All**	**Never smokers**	**Ever smokers**
	**N**, **in analysis**	6822	2901	3921
*GSTO1*	Daily ETS	−27 (−56;3)	−31 (−79 ; 16)	−28 (−66 ; 10)
	rs4925	61 (−37;49)	**67** (**6** ; **128**)^*^	−46 (−106 ; 15)
	ETS*rs4925	−**115** (−**209**;-**22**)^*^	−153 (−306 ; 0)	−72 (−192 ; 47)
*GSTO1*	Daily ETS	−25 (−55;5)	−28 (−77 ; 22)	−28 (−67 ; 11)
	rs1147611	15 (−22;51)	**56** (**5** ; **107**)^*^	−24 (−75 ; 28)
	ETS*rs1147611	−**83** (−**159**;-**7**)^*^	−122 (−247 ; 3)	−45 (−147 ; 52)
*GSTO2*	Daily ETS	−29 (−59;1)	−28 (−77 ; 22)	−34 (−73 ; 5)
	rs156697	6 (−31;43)	**57** (**5** ; **109**)^*^	−40 (−93 ; 12)
	ETS*rs156697	−59 (−137;19)	−**127** (−**252** ; -**1**)^*^	−2 (−104 ; 99)
*GSTO2*	Daily ETS	−26 (−56;3)	−29 (−78 ; 19)	−28 (−66 ; 10)
	rs156699	6 (−35;46)	44 (−12 ; 100)	−29 (−86 ; 28)
	ETS*rs156699	−**94** (−**178**;-**10**)^*^	−133 (−268 ; 2)	−56 (−165 ; 54)

The linear regression model for the whole group was adjusted for sex, age, height, weight, current, ex-smoking and packyears smoked. Consequently we stratified by smoking status (never/ever) and adjusted for the other possible confounders.

^*^p-value<0.05.

There were no significant effects after FDR correction for multiple testing.

**Table 4:** **Effects for workplace ETS exposure** (**n**/**y**), **the SNPs**, **and the interaction of *GSTO *SNPs** (**recessive model**) **with workplace ETS exposure on FEV**_**1**_

			**FEV**_**1 **_**(ml) b (95% CI)**	
**Gene**	**Variable**	**All**	**Never smokers**	**Ever smokers**
	**N**, **in analysis**	7149	3051	4098
*GSTO1*	Workplace ETS	−26 (−71 ; 20)	−51 (−126 ; 25)	−17 (−73 ; 40)
	rs4925	8 (−34 ; 50)	78 (17 ; 138)	−48 (−105 ; 9)
	ETS*rs4925	^$^-**173** (−**313** ; -**33**)^*^	^$^-**281** (−**498** ; -**64**)^*^	−84 (−268 ; 100)
*GSTO1*	Workplace ETS	−22 (−68 ; 25)	−41 (−117 ; 36)	−15 (−73 ; 43)
	rs1147611	8 (−27 ; 42)	49 (0 ; 99)^#^	−29 (−78 ; 19)
	ETS*rs1147611	^$^-**151** (−**272** ; -**31**)^*^	^$^-**286** (−**486** ; -**87**)^**^	−65 (−218 ; 87)
*GSTO2*	Workplace ETS	−21 (−67 ; 25)	−41 (−118 ; 36)	−14 (−72 ; 44)
	rs156697	8 (−28 ; 44)	50 (−1 ; 100)	−29 (−79 ; 20)
	ETS*rs156697	^$^-**170** (−**295** ; -**46**)^**^	^$^-**287** (−**486** ; -**88**)^**^	−84 (−245 ; 77)
*GSTO2*	Workplace ETS	−17 (−63 ; 28)	−41 (−117 ; 35)	−9 (−66 ; 48)
	rs156699	6 (−32 ; 45)	43 (−12 ; 98)	−26 (−79 ; 28)
	ETS*rs156699	^$^-**218** (−**349** ; -**87**)^**^	^$^-**318** (−**525**;-**111**)^**^	−140 (−311 ; 30)

The linear regression model for the whole group was adjusted for sex, age, height, weight, current, ex-smoking and packyears smoked. Consequently we stratified by smoking status (never/ever) and adjusted for the other possible confounders.

^*^p-value<0.05 ^**^p-value<0.01.

^$^significant after FDR correction for multiple testing.

### Verification of initial findings in the second sample

Population characteristics (Table 1) and genotype frequencies (Additional file 1: Table S1) were similar in the second (verification) and the first sample. Complete data was available for n = 3914, 4527 and 4702 subjects for *in utero*, daily and workplace ETS respectively. Estimates for the negative associations of *in utero*, daily and workplace ETS exposure on FEV_1_ and FEV_1_/FVC in the verification sample, were in line with associations found in sample 1, yet did not all reach statistical significance (Additional file 1: Table S7). Associations between the heterozygote genotypes for rs4925 and rs156699 with FEV_1_ and FEV_1_/FVC respectively, found in sample 1, could not be replicated in the second sample (Additional file 1: Table S3).

#### Interaction GSTO SNPs with in utero ETS

Similar to sample 1, *GSTO2* SNP rs156697 significantly interacted with *in utero* ETS exposure and was associated with a higher level of FEV_1_ in sample 2 (Table 5). The other *GSTO* SNPs consistently had effects in the similar direction, yet without reaching statistical significance.

**Table 5:** **Verification of the interaction of *GSTO *SNPs** (**recessive model**) **with different types of ETS exposure on FEV**_**1 **_**in sample 2**

			**FEV**_**1 **_**(ml) b (95% CI)**	
**Gene**	**Variable**	***In utero *ETS**	**Daily ETS**	**Workplace ETS**
	**N**, **in analysis**	3914	4527	4702
*GSTO1*	ETS	−45 (−91 ; 1)	−**41** (−**78** ; -**4**)*	−40 (−98 ; 18)
	rs4925	11 (−43 ; 65)	15 (−36 ; 66)	33 (−17 ; 83)
	ETS*rs4925	111 (−32 ; 253)	25 (−93 ; 142)	−163 (−390 ; 65)
*GSTO1*	ETS	−**49** (−**96** ; -**1**)^*^	−**40** (−**78** ; -**2**)*	−40 (−100 ; 20)
	rs1147611	4 (−40 ; 47)	21 (−21 ; 63)	26 (−15 ; 67)
	ETS*rs1147611	94 (−22 ; 211)	5 (−89 ; 98)	−87 (−254 ; 81)
*GSTO2*	ETS	−**52** (−**99** ; -**5**)^*^	−**41** (−**79** ; -**4**)*	−38 (−98 ; 21)
	rs156697	2 (−42 ; 46)	17 (−25 ; 59)	28 (−13 ; 69)
	ETS*rs156697	**119** (**0** ; **237**)^*^	17 (−79 ; 113)	−99 (−269 ; 70)
*GSTO2*	ETS	−46 (−92 ; 1)	−**40** (−**78** ; -**3**)*	−42 (−101 ; 17)
	rs156699	−9 (−58 ; 39)	0 (−46 ; 47)	10 (−35 ; 56)
	ETS*rs156699	106 (−29 ; 241)	9 (−97 ; 115)	−101 (−292 ; 91)

Effects for in utero ETS exposure (no/yes), daily ETS exposure (</≥1 hr), workplace ETS exposure (no/yes), the SNPs, and the interaction of GSTO SNPs (recessive model) with different types of ETS exposure on FEV_1_ in sample 2 (verification). The linear regression model for the whole group was adjusted for sex, age, height, weight, current, ex-smoking and packyears smoked.

^*^p-value<0.05.

#### Interaction GSTO SNPs with daily and workplace ETS exposure

Analyses in the verification sample did not show any significant interaction or trend for interaction between the *GSTO* SNPs and daily (≥1 hour) ETS exposure (Table 5). In line with findings in sample 1, there were clear interactions of the *GSTO* SNPs with workplace ETS exposure that were associated with lower level of FEV_1_, but these interactions were non-significant in sample 2 (Table 5). Full results of the (stratified) analyses in sample 2 can be found in Additional file 1: Tables S8, S9 and S10.

Overall, the directions of the interactions of *in utero* and workplace ETS exposure with the SNPs found in the second (verification) sample were in line with the first sample, but effect estimates were somewhat smaller and not always significant.

## Discussion

### Main finding

This study is the first to show that *GSTO* SNPs interact with ETS exposure on FEV_1_, findings that were significant after FDR correction for multiple testing and replicated in the second (verification) sample or showed similar directions of effects. Interestingly, interactions were in opposite directions for ETS exposure *in utero* and during adulthood.

### Results in relation to other studies

Smoking during pregnancy has been shown to reduce tidal flow-volume ratios in healthy newborn babies [4,5] and to reduce small airway flows in school age children [29]. We found no significant effect of *in utero* ETS exposure on level of FEV_1_ in adulthood in both our study samples, which does not exclude that effects might be present when studying more specifically small airway dimensions as derived in school age children [29]. In line with other studies investigating effects of ETS exposure during adulthood [6,7], we found daily and workplace ETS to be associated with lower levels of FEV_1_, and these effects were more pronounced in never smokers. Our effect estimate of a 45 ml lower FEV_1_ level with daily ETS exposure in never smokers was comparable with the 35 ml (−66 ; -4) reduced FEV_1_ level with daily ETS exposure of 1 to 4 hours in never smokers from the European Community Respiratory Health Survey (ECRHS) [7].

The homozygote mutant genotype for SNP rs156697 was not associated with the level of FEV_1_ in our sample, but there was a significant interaction with *in utero* ETS exposure that was associated with higher level of FEV_1_. This was a robust finding that remained significant after FDR correction for multiple testing and was moreover significantly replicated in the verification sample. Interactions between the other three SNPs and *in utero* ETS exposure showed clear trends for an association with higher level of FEV_1_ in both samples but only reached significance in the first sample. Interestingly, the homozygote mutant genotypes for SNPs rs4925, rs1147611, rs156699 significantly interacted with daily ETS exposure, and all SNPs (rs4925, rs1147611, rs156697, and rs156699) significantly interacted with workplace ETS exposure and were associated with lower level of FEV_1_ in the first sample. The interactions with workplace ETS exposure remained significant after FDR correction for multiple testing and showed a clear trend for interaction in the similar direction in the verification sample. These latter results support previous findings that *GSTO2* is a risk gene for lower levels of FEV_1_ and FVC [12].

How can we reconcile that exposure to (harmful) ETS *in utero* does not result in lower but higher adult level of lung function in subjects who are homozygote mutant for the *GSTO* “risk” alleles, whereas adult ETS exposure in these individuals associates with lower lung function? First, exposure to ETS *in utero* likely leads to exposure to different substances and concentrations of substances than ‘direct’ inhalation of ETS. It is conceivable that substances of ETS will be ‘filtered’ by the maternal lung and circulation, the placenta and fetal circulation. In addition, it is also conceivable that chronic ETS during pregnancy induces maternal changes that are important for lung growth. For example, it is well-known that nicotine inhaled with cigarette smoking stimulates secretion of growth hormone in humans [30]. This is particularly interesting because growth hormone has been shown to stimulate lung growth as well as lung development during the period of alveolarization [31].

Another explanation relates to exposure to ETS taking place in completely different periods of the life-span. Different biochemical and biological processes are involved in lung development *in utero*, lung growth in childhood and early adulthood, and lung function decline in adulthood. ETS may therefore cause differential and even contradictory effects in different periods of life. For example, oxidative stress *in utero* possibly does not only damage, but is additionally necessary for cell apoptosis during lung morphogenesis. A recent study showed that risk genotypes for the non-synonymous SNPs rs4925 (Ala140Asp) in *GSTO1* and rs156697 (Asn142Asp) in *GSTO2* reduce *GSTO2* expression levels, leading to accumulation of oxidative damage [32]. Increased oxidant levels may contribute to cell apoptosis and subsequently better airway branching *in utero*, with positive effects on FEV_1_ levels. This may contrast to adult life where airway branching has stopped and oxidative stress has predominantly negative effects, i.e. induced epithelial and endothelial cell damage and apoptosis that may contribute to airway wall and/or lung tissue fibrosis and subsequently a lower level of FEV_1_. Obviously, different biological processes and pathways underlie the differential effects of ETS *in utero* versus later in life. However, all given explanations are speculative and merit further research.

Generally we found that the interactions between daily and workplace ETS exposure and the *GSTO* SNPs were more pronounced in the never smokers. For *in utero* ETS exposure this difference was less evident. These findings might suggest that among ever smokers the effects are somewhat overruled by the effects of personal smoking, that may damage the lung by similar mechanisms yet with higher doses. We were not able to test if the interaction between *GSTO* SNPs and ETS was significantly different between the never and ever smokers since we did not have enough study power for testing this three-way interaction between smoking status and *GSTO* SNPs and ETS.

We did not find consistent significant interaction effects of the *GSTO* SNPs and ETS exposure on FEV_1_/FVC. Since the interactions were negatively associated with FEV_1_ but not with FEV_1_/FVC, in an additional analysis we investigated effects on FVC. In line with effects on FEV_1_, all four *GSTO* SNPs interacted positively with *in utero* ETS exposure and negatively with workplace ETS exposure on FVC level. Daily ETS exposure interacted negatively with the *GSTO* SNPs on FVC, but these associations were not significant. These findings suggest restrictive rather than obstructive effects on lung function.

### Strengths and limitations

The extensive standardized characterization of the LifeLines population and the large sample size provided the unique opportunity to investigate gene-by-environment interactions. A major strength was the inclusion of a large verification sample that is very similar to the discovery sample. Since the verification sample was somewhat smaller than the identification sample, its power might have been too low to replicate the significant associations, but we observed clear trends in similar directions. Haplotype analysis did not provide additional information and was therefore not shown. In the current study we have adjusted for traditional covariates related to level of lung function. Additional adjustment for highest obtained level of education, as proxy for socio-economic status, ever having had a cardiovascular event or bronchodilator use did not change our results.

A limitation of our study might be the cross-sectional design with rather crude assessment of ETS exposure, without data on lifetime exposure and quantitative measurement of workplace exposure. Objective measures of exposure to environmental tobacco smoking such as cotinine levels in serum or urine were unfortunately not available. However, the exact questions as defined in the ECRHS surveys were used in our study, and these questions were validated in an Italian subsample of the ECRHS. The question about the number of hours that a person is exposed to other people’s tobacco smoke showed a modest correlation with serum cotinine levels, with a clear dose–response effect between the number of hours and cotinine levels [33]. Notwithstanding this, it should be acknowledged that using self-reports may lead to recall bias, i.e. people experiencing respiratory illness are more likely to recall and report ETS exposure.

## Conclusions

Our data show that polymorphisms in *GSTO* genes, involved in oxidative stress pathways and detoxification of xenobiotic substances interact with ETS exposure both *in utero* and in adulthood and significantly affect the level of FEV_1_.

## Abbreviations

ETS: Environmental tobacco smoke; SNP: Single nucleotide polymorphism; GST: Glutathione S-Transferase; GSTO: Glutathione S-Transferase Omega; COPD: Chronic obstructive pulmonary disease; CF: Cystic fibrosis; FEV1: Forced expiratory volume in one second; FVC: Forced vital capacity; FDR: False discovery rate.

## Competing interests

The University of Groningen has received money for NHT ten Hacken regarding an unrestricted educational grant for research from Chiesi, GSK, and Boehringer. The University of Groningen has received money for DS Postma regarding an unrestricted educational grant for research from AstraZeneca, Chiesi, GSK. Travel of to ERS and/or ATS has been partially funded by AstraZeneca, Chiesi, GSK, Nycomed. Fees for consultancies were given to the University of Groningen by AZ, Boehringer Ingelheim, Chiesi, GSK, Nycomed and TEVA. None of the other authors has conflicts of interest related to this work.

## Authors’ contributions

KdJ participated in the study design, analyzed the data and wrote the manuscript. HMB, DSP and JMV obtained funding, determined the study design, participated in the analysis and interpretation of data, and critically supervised writing of the manuscript. NTH has significantly contributed to the results, the discussion and writing of the manuscript. All authors read and approved the final manuscript.

## Supplementary Material

Additional file 1: Figure S1 LD plot showing R^2^ between genotyped (rs1147611 and rs156699) and imputed (rs4925 and rs156697) *GSTO1* and *GSTO2* SNPs in sample 1. **Table S1.** Genotype frequencies and minor allele frequency (MAF) for the four tagging SNPs in the *GSTO1*-*2* cluster in samples 1 and 2. **Table S2.** Associations between ETS exposure and lung function level in sample 1. **Table S3.** Associations between genotypes and lung function in samples 1 and 2. **Table S4.** Effects for *in utero* ETS exposure (no/yes), the SNPs, and the interaction of *GSTO* SNPs (recessive model) with *in utero* ETS exposure on FEV_1_/FVC (%) in sample 1. **Table S5.** Effects for daily ETS exposure (</≥1hr), the SNPs, and the interaction of *GSTO* SNPs (recessive model) with daily ETS exposure on FEV_1_/FVC (%) in sample 1. **Table S6.** Effects for workplace ETS exposure (n/y), the SNPs, and the interaction of *GSTO* SNPs (recessive model) with workplace ETS exposure on FEV_1_/FVC (%) in sample 1. **Table S7.** Verification: Associations between ETS exposure and lung function level (FEV_1_ and FEV_1_/FVC (%)) in sample 2. **Table S8.** Verification: Effects for *in utero* ETS exposure (no/yes), the SNPs, and the interaction of *GSTO* SNPs (recessive model) with *in utero* ETS exposure on FEV_1_ in sample 2. **Table S9.** Verification: Effects for daily ETS exposure (</≥1hr), the SNPs, and the interaction of *GSTO* SNPs (recessive model) with daily ETS exposure on FEV_1_ in sample 2. **Table S10.** Verification: Effects for workplace ETS exposure (n/y), the SNPs, and the interaction of *GSTO* SNPs (recessive model) with workplace ETS exposure on FEV_1_ in sample 2. Click here for file
