# Supplementary material for: GST-omega genes interact with environmental tobacco smoke on adult level of lung function
Source: Respir Res. 2013 Aug 9;14(1):83. doi: 10.1186/1465-9921-14-83 (PMC3751364; doi:10.1186/1465-9921-14-83)
Supplement: Additional file 1: Figure S1 — LD plot showing R2 between genotyped (rs1147611 and rs156699) and imputed (rs4925 and rs156697) GSTO1 and GSTO2 SNPs in sample 1. Table S1. Genotype frequencies and minor allele frequency (MAF) for the four tagging SNPs in the GSTO1-2 cluster in samples 1 and 2. Table S2. Associations between ETS exposure and lung function level in sample 1. Table S3. Associations between genotypes and lung function in samples 1 and 2. Table S4. Effects for in utero ETS exposure (no/yes), the SNPs, and the interaction of GSTO SNPs (recessive model) with in utero ETS exposure on FEV1/FVC (%) in sample 1. Table S5. Effects for daily ETS exposure (</≥1hr), the SNPs, and the interaction of GSTO SNPs (recessive model) with daily ETS exposure on FEV1/FVC (%) in sample 1. Table S6. Effects for workplace ETS exposure (n/y), the SNPs, and the interaction of GSTO SNPs (recessive model) with workplace ETS exposure on FEV1/FVC (%) in sample 1. Table S7. Verification: Associations between ETS exposure and lung function level (FEV1 and FEV1/FVC (%)) in sample 2. Table S8. Verification: Effects for in utero ETS exposure (no/yes), the SNPs, and the interaction of GSTO SNPs (recessive model) with in utero ETS exposure on FEV1 in sample 2. Table S9. Verification: Effects for daily ETS exposure (</≥1hr), the SNPs, and the interaction of GSTO SNPs (recessive model) with daily ETS exposure on FEV1 in sample 2. Table S10. Verification: Effects for workplace ETS exposure (n/y), the SNPs, and the interaction of GSTO SNPs (recessive model) with workplace ETS exposure on FEV1 in sample 2. [file 1465-9921-14-83-S1.doc]

**Original Research**

*GST-omega* genes modify effects of environmental tobacco smoke on adult level of lung function

Kim de Jong, H. Marike Boezen, Nick H. T. ten Hacken, Dirkje S. Postma, Judith M. Vonk.

**Supplementary data: Figure 1 and Tables 1-10**

Supplementary figure 1. LD plot showing R^2^ between genotyped (rs1147611 and rs156699) and imputed (rs4925 and rs156697) *GSTO1* and *GSTO2* SNPs with MAF ≥ 0.1 and HW-equilibrium p-value > 0.05, in n = 8,128 subjects included in sample 1.


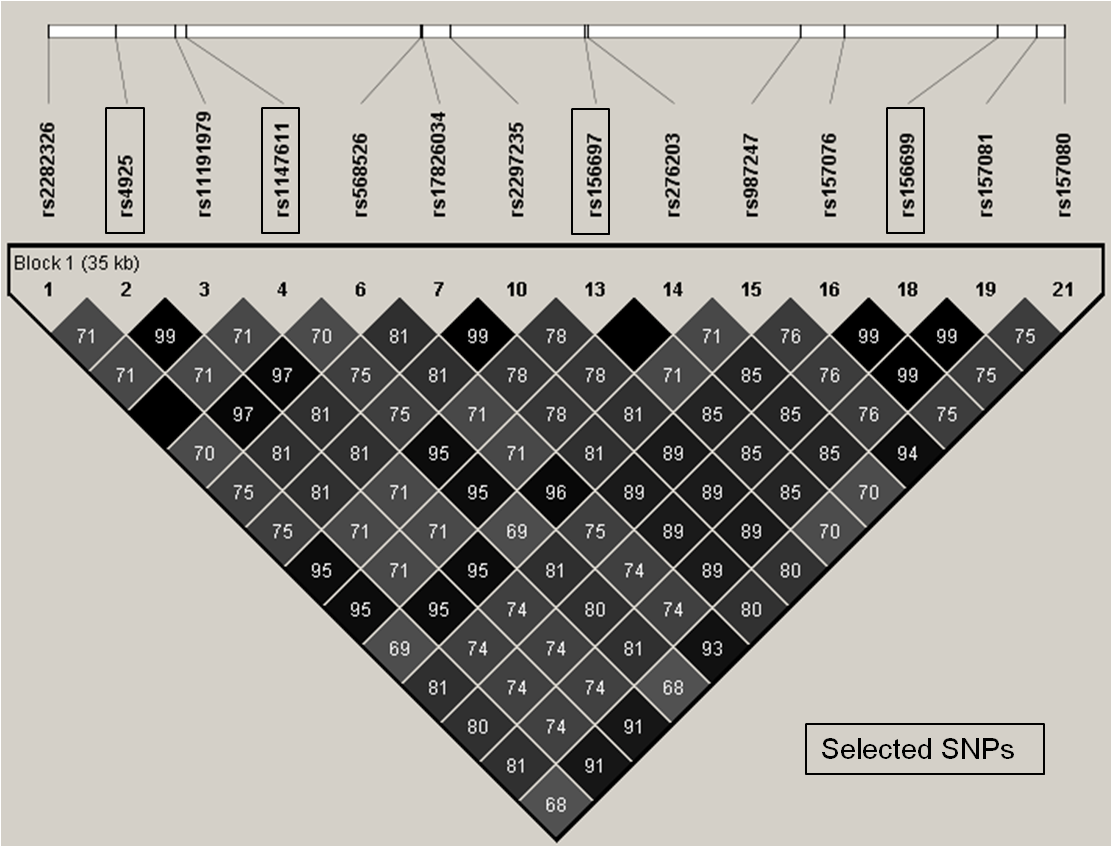


Supplementary table 1. Genotype frequencies and minor allele frequency (MAF) for the four tagging SNPs in the *GSTO1-2* cluster in n = 8,128 subjects included in sample 1 and n = 5,308 subjects in sample 2 (verification).

|  | **Sample 1** | | | | | | | | | | | | | |
| --- | --- | --- | --- | --- | --- | --- | --- | --- | --- | --- | --- | --- | --- | --- |
| **Gene** | | **SNP** |  | **Wild type** |  | **Heterozygote** |  | **Homozygote**  **mutant** |  | **MAF** |  | **Major**^#^ |  | **Minor**^#^ |
| *GSTO1* | | rs4925 |  | 4060 (50%) |  | 3345 (41%) |  | 723 (9%) |  | 0.30 |  | C |  | A |
| *GSTO1* | | rs1147611 |  | 3329 (41%) |  | 3688 (45%) |  | 1111 (14%) |  | 0.36 |  | G |  | T |
| *GSTO2* | | rs156697 |  | 3419 (42%) |  | 3663 (45%) |  | 1046 (13%) |  | 0.35 |  | A |  | G |
| *GSTO2* | | rs156699 |  | 3744 (46%) |  | 3508 (43%) |  | 876 (11%) |  | 0.32 |  | A |  | G |
|  | **Sample 2** | | | | | | | | | | | | | |
| **Gene** | | **SNP** |  | **Wild type** |  | **Heterozygote** |  | **Homozygote**  **mutant** |  | **MAF** |  | **Major**^#^ |  | **Minor**^#^ |
| *GSTO1* | | rs4925 |  | 2642 (50%) |  | 2202 (42%) |  | 464 (9%) |  | 0.29 |  | C |  | A |
| *GSTO1* | | rs1147611 |  | 2122 (40%) |  | 2442 (46%) |  | 744 (14%) |  | 0.37 |  | G |  | T |
| *GSTO2* | | rs156697 |  | 2157 (41%) |  | 2435 (46%) |  | 716 (14%) |  | 0.36 |  | A |  | G |
| *GSTO2* | | rs156699 |  | 2394 (45%) |  | 2336 (44%) |  | 578 (11%) |  | 0.33 |  | A |  | G |

^#^ = Major (wild type) and minor allele (modeled as risk allele) respectively

Supplementary table 2. Associations between ETS exposure and lung function level in the n = 8,128 subjects included in sample 1. Associations were assessed by linear regression models, all adjusted for sex, age, height, weight, current, ex-smoking and packyears smoked. Additionally the model was stratified by smoking status (never/ever), and adjusted for the other confounders.

|  |  |  |  | **FEV_1_ (ml)**  **b (95% CI)** |  |  |
| --- | --- | --- | --- | --- | --- | --- |
| **ETS exposure** |  | **All** |  | **Never smokers** |  | **Ever smokers** |
| *In utero* |  | -19 (-54 ; 17) |  | -6 (61 ; 48) |  | -31 (-78 ; 15) |
| Daily |  | **-37 (-65 ; -8)**^*^ |  | -45 (-91 ; 0)^#^ |  | -34 (-70 ; 3) |
| At work |  | **-43 (-86 ; 0)**^*^ |  | **-82 (-153 ; -11**)^*^ |  | -25 (-79 ; 29) |
|  |  |  |  | **FEV_1_/FVC**  **b (95% CI)** |  |  |
| **ETS exposure** |  | **All** |  | **Never smokers** |  | **Ever smokers** |
| *In utero* |  | **-0.6 (-1.1 ; -0.1)**^*^ |  | -0.6 (-1.4 ; 0.1) |  | -0.6 (-1.3 ; 0.1) |
| Daily |  | -0.3 (-0.7 ; 0.1) |  | 0 (-0.7 ; 0.6) |  | -0.4 (-1.0 ; 0.1) |
| At work |  | **-0.6 (-1.2 ; 0)^*^** |  | -0.4 (-1.4 ; 0.5) |  | -0.7 (-1.5 ; 0.1) |

^#^p = 0.051; ^*^p<0.05

Supplementary table 3. Associations between genotypes and lung function, adjusted for sex, age, height, weight, current smoking, ex-smoking and packyears smoked in sample 1 and sample 2 (verification). The wild type genotype was used as reference category.

|  | |  | |  |  | **Sample 1 (n=8,128)** | | |  | **Sample 2 (n=5,308)** | | |
| --- | --- | --- | --- | --- | --- | --- | --- | --- | --- | --- | --- | --- |
| **SNPs** |  | | **Alleles** | |  | **FEV_1_ (ml)**  **b (95% CI)** |  | **FEV_1_/FVC**  **b (95% CI)** |  | **FEV_1_ (ml)**  **b (95% CI)** |  | **FEV_1_/FVC**  **b (95% CI)** |
| **rs4925** |  | | **CA** | |  | **23 (0 ; 45)^*^** |  | 0.3 (-0.8 ; 0.6) |  | -7 (-34 ; 20) |  | 0.2 (-0.2 ; 0.5) |
|  |  | | **AA** | |  | -7 (-45 ; 32) |  | -0.1 (-0.7 ; 0.4) |  | 24 (-23 ; 70) |  | 0.2 (-0.2 ; 1.1) |
| **rs1147611** |  | | **GT** | |  | 11 (-12 ; 34) |  | 0.2 (-0.1 ; 0.5) |  | -1 (-29 ; 26) |  | -0.1 (-0.5 ; 0.3) |
|  |  | | **TT** | |  | -2 (-36 ; 31) |  | -0.2 (-0.7 ; 0.3) |  | 22 (-18 ; 62) |  | 0.5 (-0.1 ; 1.0) |
| **rs156697** |  | | **AG** | |  | 11 (-12 ; 34) |  | 0.2 (-0.9 ; 0.6) |  | 1 (-27 ; 28) |  | 0 (0 ; 0.4) |
|  |  | | **GG** | |  | -5 (-39 ; 29) |  | -0.2 (-0.7 ; 0.3) |  | 23 (-17 ; 63) |  | 0.5 (-0.1 ; 1.0) |
| **rs156699** |  | | **AG** | |  | 21 (-2 ; 43) |  | **0.3 (0 ; 0.7)^*^** |  | -8 (-36 ; 19) |  | 0 (-0.4 ; 0.4) |
|  |  | | **GG** | |  | -8 (-44 ; 29) |  | -0.4 (-0.9 ; 0.2) |  | 2 (-41 ; 45) |  | 0.3 (-0.3 ; 1.0) |

^*^p-value<0.05

Supplementary table 4. Effects for *in utero* ETS exposure (no/yes), the SNPs, and the interaction of *GSTO* SNPs (recessive model) with *in utero* ETS exposure on FEV_1_/FVC (%). The linear regression model for the whole group was adjusted for sex, age, height, weight, current, ex-smoking and packyears smoked. Consequently we stratified by smoking status (never/ever) and adjusted for the other possible confounders.

|  |  |  |  |  | **FEV_1_/FVC**  **b (95% CI)** |  |  |
| --- | --- | --- | --- | --- | --- | --- | --- |
|  |  |  |  |  |  |  |  |
| **Gene** | **SNPs** |  | **All** |  | **Never smokers** |  | **Ever smokers** |
|  | **N, in analysis** |  | 6003 |  | 2576 |  | 3427 |
|  |  |  |  |  |  |  |  |
| *GSTO1* | *In utero* ETS |  | **-0.6 (-1.0 ; -0.1)^*^** |  | -0.7 (-1.5 ; 0.1) |  | -0.5 (-1.2 ; 0.2) |
|  | rs4925 |  | -0.3 (-0.9 ; 0.3) |  | -0.3 (-1.2 ; 0.6) |  | -0.3 (-1.2 ; 0.5) |
|  | ETS*rs4925 |  | -0.4 (-2.2 ; 1.4) |  | 0.1 (-2.0 ; 3.0) |  | -1.2 (-3.8 ; 1.4) |
|  |  |  |  |  |  |  |  |
| *GSTO1* | *In utero* ETS |  | **-0.7 (-1.2 ; -0.1)^*^** |  | -0.7 (-1.5 ; 0.1) |  | -0.6 (-1.3 ; 0.1) |
|  | rs1147611 |  | -0.4 (0.9 ; 0.1) |  | -0.3 (-1.0 ; 0.5) |  | -0.6 (-1.3 ; 0.2) |
|  | ETS*rs1147611 |  | 0.3 (-1.2 ; 1.8) |  | 0.6 (-1.6 ; 2.8) |  | 0.2 (-1.9 ; 2.3) |
|  |  |  |  |  |  |  |  |
| *GSTO2* | *In utero* ETS |  | **-0.7 (-1.2 ; -0.1)^*^** |  | -0.7 (-1.5 ; 0.1) |  | -0.6 (-1.3 ; 0.1) |
|  | rs156697 |  | -0.5 (-1.0 ; 0) |  | -0.4 (-1.1 ; 0.4) |  | -0.6 (-1.4 ; 0.1) |
|  | ETS*rs156697 |  | 0.4 (-1.2 ; 1.9) |  | 0.5 (-1.8 ; 2.7) |  | 0.3 (-1.9 ; 2.4) |
|  |  |  |  |  |  |  |  |
| *GSTO2* | *In utero* ETS |  | **-0.6 (-1.1 ; 0)^*^** |  | -0.7 (-1.5 ; 0.1) |  | -0.5 (-1.2 ; 0.2) |
|  | rs156699 |  | **-0.6 (-1.1 ; 0)^*^** |  | -0.5 (-1.3 ; 0.3) |  | -0.6 (-1.4 ; 0.2) |
|  | ETS*rs156699 |  | -0.5 (-2.2 ; 1.2) |  | 0.5 (-2.0 ; 2.9) |  | -1.1 (-3.4 ; 1.2) |

^*^p-value<0.05

Supplementary table 5. Effects for daily ETS exposure (</≥1hr), the SNPs, and the interaction of *GSTO* SNPs (recessive model) with daily ETS exposure on FEV_1_/FVC (%). The linear regression model for the whole group was adjusted for sex, age, height, weight, current, ex-smoking and packyears smoked. Consequently we stratified by smoking status (never/ever) and adjusted for the other possible confounders.

|  |  |  |  |  | **FEV_1_/FVC**  **b (95% CI)** |  |  |
| --- | --- | --- | --- | --- | --- | --- | --- |
|  |  |  |  |  |  |  |  |
| **Gene** | **SNPs** |  | **All** |  | **Never smokers** |  | **Ever smokers** |
|  | **N, in analysis** |  | 6822 |  | 2901 |  | 3921 |
|  |  |  |  |  |  |  |  |
| *GSTO1* | Daily ETS |  | -0.2 (-0.6 ; 0.2) |  | 0.1 (-0.5 ; 0.8) |  | -0.4 (-0.9 ; 0.2) |
|  | rs4925 |  | 0 (-0.6 ; 0.6) |  | 0.3 (-0.6 ; 1.1) |  | -0.2 (-1.1 ; 0.7) |
|  | ETS*rs4925 |  | -1.1 (-2.4 ; 0.2) |  | -1.7 (-3.9 ; 0.4) |  | -0.7 (-2.4 ; 1.0) |
|  |  |  |  |  |  |  |  |
| *GSTO1* | Daily ETS |  | -0.2 (-0.6 ; 0.2) |  | 0.1 (-0.6 ; 0.8) |  | -0.3 (-0.9 ; 0.2) |
|  | rs1147611 |  | -0.1 (-0.6 ; 0.5) |  | 0.1 (-0.6 ; 0.8) |  | -0.1 (-0.9 ; 0.6) |
|  | ETS*rs1147611 |  | -0.7 (-1.8 ; 0.4) |  | -0.7 (-2.4 ; 1.1) |  | -0.7 (-2.1 ; 0.7) |
|  |  |  |  |  |  |  |  |
| *GSTO2* | Daily ETS |  | -0.2 (-0.7 ; 0.2) |  | 0.1 (-0.6 ; 0.8) |  | 0.1 (-0.5 ; 0.8) |
|  | rs156697 |  | -0.1 (-0.7 ; 0.4) |  | 0 (-0.7 ; 0.7) |  | 0.1 (-0.7 ; 0.9)  11-1.3 (-0.3 ; 0.6) |
|  | ETS*rs156697 |  | -0.6 (-1.7 ; 0.5) |  | -0.7 (-2.4 ; 1.1) |  | -1.3 (-0.3 ; 0.6) |
|  |  |  |  |  |  |  |  |
| *GSTO2* | Daily ETS |  | -0.2 (-0.6 ; 0.2) |  | -0.4 (-0.9 ; 0.2) |  | -0.3 (-0.9 ; 0.2) |
|  | rs156699 |  | -0.2 (-0.7 ; 0.4) |  | -0.2 (-1.0 ; 0.5) |  | -0.4 (-1.2 ; 0.4) |
|  | ETS*156699 |  | -1.0 (-2.2 ; 0.1) |  | -0.5 (-2.0 ; 0.9) |  | -0.8 (-2.4 ; 0.8) |

Supplementary table 6. Effects for workplace ETS exposure (n/y), the SNPs, and the interaction of *GSTO* SNPs (recessive model) with workplace ETS exposure on FEV_1_/FVC (%). The linear regression model for the whole group was adjusted for sex, age, height, weight, current, ex-smoking and packyears smoked. Consequently we stratified by smoking status (never/ever) and adjusted for the other possible confounders.

|  |  |  |  |  | **FEV_1_/FVC**  **b (95% CI)** |  |  |
| --- | --- | --- | --- | --- | --- | --- | --- |
|  |  |  |  |  |  |  |  |
| **Gene** | **SNPs** |  | **All** |  | **Never smokers** |  | **Ever smokers** |
|  | **N, in analysis** |  | 7149 |  | 3051 |  | 4098 |
|  |  |  |  |  |  |  |  |
| *GSTO1* | Workplace ETS |  | -0.6 (-1.2 ; 0.1) |  | -0.2 (-1.3 ; 0.8) |  | -0.7 (-1.5 ; 0.1) |
|  | rs4925 |  | -0.3 (-0.9 ; 0.3) |  | 0.1 (-0.7 ; 0.9) |  | -0.6 (-1.4 ; 0.3) |
|  | ETS*rs4925 |  | -0.5 (-2.5 ; 1.5) |  | -1.8 (-4.8 ; 1.2) |  | 0.4 (-2.3 ; 3.0) |
|  |  |  |  |  |  |  |  |
| *GSTO1* | Workplace ETS |  | -0.5 (-1.1 ; 0.2) |  | -0.1 (-1.1 ; 1.0) |  | -0.7 (-1.6 ; 0.1) |
|  | rs1147611 |  | -0.2 (-0.7 ; 0.3) |  | 0.1 (-0.6 ; 0.8) |  | -0.5 (-1.2 ; 0.2) |
|  | ETS*rs1147611 |  | -0.8 (-2.5 ; 0.9) |  | -2.6 (-5.3 ; 0.1) |  | 0.2 (-2.0 ; 2.4) |
|  |  |  |  |  |  |  |  |
| *GSTO2* | Workplace ETS |  | -0.5 (-1.1 ; 0.2) |  | -0.1 (-1.1 ; 1.0) |  | -0.7 (-1.5 ; 0.2) |
|  | rs156697 |  | -0.3 (-0.8 ; 0.2) |  | 0 (-0.7 ; 0.7) |  | -0.5 (-1.2 ; 0.2) |
|  | ETS*rs156697 |  | -1.0 (-2.7 ; 0.8) |  | -2.5 (-5.3 ; 0.2) |  | 0 (-2.3 ; 2.4) |
|  |  |  |  |  |  |  |  |
| *GSTO2* | Workplace ETS |  | -0.5 (-1.1 ; 0.2) |  | -0.1 (-1.2 ; 0.9) |  | -0.7 (-1.5 ; 0.2) |
|  | rs156699 |  | -0.4 (-0.9 ; 0.2) |  | 0 (-0.7 ; 0.8) |  | -0.7 (-1.5 ; 0.1) |
|  | ETS*rs156699 |  | -0.9 (-2.8 ; 0.9) |  | -2.3 (-5.1 ; 0.6) |  | 0 (-2.5 ; 2.5) |

Supplementary table 7. Verification: Associations between ETS exposure and lung function level (FEV_1_ and FEV_1_/FVC (%)) in the n = 5,308 subjects included in sample 2. Associations were assessed by linear regression models, all adjusted for sex, age, height, weight, current, ex-smoking and packyears smoked. Additionally the model was stratified by smoking status (never/ever) and adjusted for the other confounders.

|  |  |  |  | **FEV_1_ (ml)**  **b (95% CI)** |  |  |
| --- | --- | --- | --- | --- | --- | --- |
| **ETS exposure** |  | **All** |  | **Never smokers** |  | **Ever smokers** |
| *In utero* |  | -34 (-78 ; 10) |  | -35 (-103 ; 33) |  | -39 (-97 ; 19) |
| Daily |  | **-39 (-75 ; -4)^*^** |  | -25 (-83 ; 34) |  | **-51 (-11 ; -7)^*^** |
| At work |  | -51 (-107 ; 5) |  | -87 (-181 ; 6) |  | -35 (-106 ; 36) |
|  |  |  |  | **FEV_1_/FVC (%)**  **b (95% CI)** |  |  |
| **ETS exposure** |  | **All** |  | **Never smokers** |  | **Ever smokers** |
| *In utero* |  | **-0.7 (-1.4 ; -0.1)^*^** |  | -0.5 (-1.4 ; 0.5) |  | **-0.9 (-1.7 ; 0)^*^** |
| Daily |  | -0.5 (-1.0 ; 0.1) |  | 0.2 (-0.6 ; 1.0) |  | **-0.8 (-1.4 ; -0.1)^*^** |
| At work |  | -0.7 (-1.5 ; 0.1) |  | -0.9 (-2.2 ; 0.4) |  | -0.4 (-1.5 ; 0.6) |

^*^p-value<0.05

Supplementary table 8. Verification: Effects for *in utero* ETS exposure (no/yes), the SNPs, and the interaction of *GSTO* SNPs (recessive model) with *in utero* ETS exposure on FEV_1_ in sample 2 (verification). The linear regression model for the whole group was adjusted for sex, age, height, weight, current, ex-smoking and packyears smoked. Consequently we stratified by smoking status (never/ever) and adjusted for the other possible confounders.

|  |  |  |  |  | **FEV_1_ (ml)**  **b (95% CI)** |  |  |
| --- | --- | --- | --- | --- | --- | --- | --- |
|  |  |  |  |  |  |  |  |
| **Gene** | **SNPs** |  | **All** |  | **Never smokers** |  | **Ever smokers** |
|  | **N, in analysis** |  | 3914 |  | 1713 |  | 2201 |
|  |  |  |  |  |  |  |  |
| *GSTO1* | *In utero* ETS |  | -45 (-91 ; 1) |  | -47 (-117 ; 24) |  | -49 (-110 ; 12) |
|  | rs4925 |  | 11 (-43 ; 65) |  | 1 (-79 ; 81) |  | 18 (-55 ; 91) |
|  | ETS*rs4925 |  | 111 (-32 ; 253) |  | 123 (-102 ; 347) |  | 106 (-80 ; 291) |
|  |  |  |  |  |  |  |  |
| *GSTO1* | *In utero* ETS |  | **-49 (-96 ; -1)**^*^ |  | -69 (-142 ; 4) |  | -43 (-105 ; 20) |
|  | rs1147611 |  | 4 (-40 ; 47) |  | 5 (-59 ; 70) |  | 1 (-58 ; 60) |
|  | ETS*rs1147611 |  | 94 (-22 ; 211) |  | **214 (33 ; 394)^*^** |  | 28 (-125 ; 181) |
|  |  |  |  |  |  |  |  |
| *GSTO2* | *In utero* ETS |  | **-52 (-99 ; -5)**^*^ |  | -67 (-140 ; 6) |  | -49 (-111 ; 14) |
|  | rs156697 |  | 2 (-42 ; 46) |  | 5 (-60 ; 71) |  | -1 (-61 ; 59) |
|  | ETS*rs156697 |  | **119 (0 ; 237)**^*^ |  | **208 (25 ; 391)^*^** |  | 69 (-86 ; 224) |
|  |  |  |  |  |  |  |  |
| *GSTO2* | *In utero* ETS |  | -46 (-92 ; 1) |  | -52 (-123 ; 19) |  | -47 (-108 ; 14) |
|  | rs156699 |  | -9 (-58 ; 39) |  | 1 (-71 ; 74) |  | -17 (-83 ; 48) |
|  | ETS*rs156699 |  | 106 (-29 ; 241) |  | 180 (-42 ; 403) |  | 73 (-99 ; 244) |

^*^p-value<0.05

|  |  |  |  |  | **FEV_1_ (ml)**  **b (95% CI)** |  |  |
| --- | --- | --- | --- | --- | --- | --- | --- |
|  |  |  |  |  |  |  |  |
| **Gene** | **SNPs** |  | **All** |  | **Never smokers** |  | **Ever smokers** |
|  | **N, in analysis** |  | 4527 |  | 1898 |  | 2629 |
|  |  |  |  |  |  |  |  |
| *GSTO1* | Daily ETS |  | -41 (-78 ; -4) |  | -35 (-97 ; 26) |  | -50 (-97 ; -3) |
|  | rs4925 |  | 15 (-36 ; 66) |  | 1 (-75 ; 77) |  | 24 (-45 ; 93) |
|  | ETS*rs4925 |  | 25 (-93 ; 142) |  | 123 (-83 ; 328) |  | -15 (-161 ; 131) |
|  |  |  |  |  |  |  |  |
| *GSTO1* | Daily ETS |  | -40 (-78 ; -2) |  | -36 (-98 ; 27) |  | -49 (-97 ; -1) |
|  | rs1147611 |  | 21 (-21 ; 63) |  | 25 (-36 ; 85) |  | 15 (-43 ; 72) |
|  | ETS*rs1147611 |  | 5 (-89 ; 98) |  | 92 (-82 ; 266) |  | -15 (-130 ; 100) |
|  |  |  |  |  |  |  |  |
| *GSTO2* | Daily ETS |  | **-41 (-79 ; -4)**^*^ |  | -32 (-94 ; 30) |  | -52 (-101 ; -4) |
|  | rs156697 |  | 17 (-25 ; 59) |  | 23 (-38 ; 84) |  | 11 (-47 ; 69) |
|  | ETS*rs156697 |  | 17 (-79 ; 113) |  | 64 (-116 ; 243) |  | 10 (-107 ; 127) |
|  |  |  |  |  |  |  |  |
| *GSTO2* | Daily ETS |  | **-40 (-78 ; -3)**^*^ |  | -35 (-97 ; 26) |  | -49 (-96 ; -1) |
|  | rs156699 |  | 0 (-46 ; 47) |  | -10 (-79 ; 59) |  | 5 (-58 ; 68) |
|  | ETS*156699 |  | 9 (-97 ; 115) |  | 111 (-85 ; 307) |  | -24 (-154 ; 106) |

Supplementary table 9. Verification: Effects for daily ETS exposure (</≥1hr), the SNPs, and the interaction of *GSTO* SNPs (recessive model) with daily ETS exposure on FEV_1_ in sample 2 (verification). The linear regression model for the whole group was adjusted for sex, age, height, weight, current, ex-smoking and packyears smoked. Consequently we stratified by smoking status (never/ever) and adjusted for the other possible confounders.

^*^p-value<0.05

Supplementary table 10. Verification: Effects for workplace ETS exposure (n/y), the SNPs, and the interaction of *GSTO* SNPs (recessive model) with workplace ETS exposure on FEV_1_ in sample 2 (verification). The linear regression model for the whole group was adjusted for sex, age, height, weight, current, ex-smoking and packyears smoked. Consequently we stratified by smoking status (never/ever) and adjusted for the other possible confounders.

|  |  |  |  |  | **FEV_1_ (ml)**  **b (95% CI)** |  |  |
| --- | --- | --- | --- | --- | --- | --- | --- |
|  |  |  |  |  |  |  |  |
| **Gene** | **SNPs** |  | **All** |  | **Never smokers** |  | **Ever smokers** |
|  | **N, in analysis** |  | 4702 |  | 2003 |  | 2699 |
|  |  |  |  |  |  |  |  |
| *GSTO1* | Workplace ETS |  | -40 (-98 ; 18) |  | -90 (-186 ; 6) |  | -17 (-91 ; 56) |
|  | rs4925 |  | 33 (-17 ; 83) |  | 21 (-54 ; 96) |  | 42 (-24 ; 109) |
|  | ETS*rs4925 |  | -163 (-390 ; 65) |  | 57 (-339 ; 111) |  | -258 (-538 ; 23) |
|  |  |  |  |  |  |  |  |
| *GSTO1* | Workplace ETS |  | -40 (-100 ; 20) |  | -87 (-184 ; 11) |  | -17 (-94 ; 59) |
|  | rs1147611 |  | 26 (-15 ; 67) |  | 32 (-29 ; 92) |  | 21 (-34 ; 76) |
|  | ETS*rs1147611 |  | -87 (-254 ; 81) |  | -26 (-191 ; 139) |  | -118 (-316 ; 80) |
|  |  |  |  |  |  |  |  |
| *GSTO2* | Workplace ETS |  | -38 (-98 ; 21) |  | -83 (-180 ; 14) |  | -17 (-94 ; 60) |
|  | rs156697 |  | 28 (-13 ; 69) |  | 31 (-30 ; 93) |  | 25 (-31 ; 80) |
|  | ETS*rs156697 |  | -99 (-269 ; 70) |  | -39 (-402 ; 324) |  | -122 (-320 ; 76) |
|  |  |  |  |  |  |  |  |
| *GSTO2* | Workplace ETS |  | -42 (-101 ; 17) |  | -88 (-183 ; 8) |  | -19 (-94 ; 56) |
|  | rs156699 |  | 10 (-35 ; 56) |  | 3 (-66 ; 72) |  | 16 (-44 ; 76) |
|  | ETS*rs156699 |  | -101 (-292 ; 91) |  | 7 (-432 ; 445) |  | -139 (-359 ; 81) |
